# Essays on Asylums for Persons of Unsound Mind

**Published:** 1853-09

**Authors:** 


*Essays on Asylums for Persons of Unsound Mind.* By JOHN M. GALT, M. D., Sup't of Eastern Lunatic Asylum of Virginia.

Dr. Galt, in these two essays, endorses the human treatment of the insane now so generally adopted. He urges the utility of reading, and recreation, on their shattered minds, and employs all those humanizing means, which always mitigate, and frequently remove insanity. S. B. H.

